# Overview of infectious complications among CAR T- cell therapy recipients

**DOI:** 10.3389/fonc.2024.1398078

**Published:** 2024-07-03

**Authors:** Swarn Arya, Zainab Shahid

**Affiliations:** ^1^ Infectious Disease Service, Memorial Sloan Kettering Cancer Center, New York, NY, United States; ^2^ Department of Medicine, Weill Cornell School of Medicine, New York, NY, United States

**Keywords:** infectious complications, chimeric antigen receptor T-cell therapy, infection management, immunoglobulin replacement therapy, vaccinations

## Abstract

Chimeric antigen receptor-modified T cell (CAR T-cell) therapy has revolutionized the management of hematological malignancies. In addition to impressive malignancy-related outcomes, CAR T-cell therapy has significant toxicity-related adverse events, including cytokine release syndrome (CRS), immune effector cell associated neurotoxicity syndrome (ICANS), immune effector cell-associated hematotoxicity (ICAHT), and opportunistic infections. Different CAR T-cell targets have different epidemiology and risk factors for infection, and these targets result in different long-term immunodeficiency states due to their distinct on-target and off- tumor effects. These effects are exacerbated by the use of multimodal immunosuppression in the management of CRS and ICANS. The most effective course of action for managing infectious complications involves determining screening, prophylactic, and monitoring strategies and understanding the role of immunoglobulin replacement and re-vaccination strategies. This involves considering the nature of prior immunomodulating therapies, underlying malignancy, the CAR T-cell target, and the development and management of related adverse events. In conclusion, we now have an increasing understanding of infection management for CAR T-cell recipients. As additional effector cells and CAR T-cell targets become available, infection management strategies will continue to evolve.

## 1 Introduction

The chimeric antigen receptor-modified T cell (CAR T-cell) therapy field has rapidly expanded since the US Food and Drug Administration (FDA) first approved CD19-targeted CAR T-cells for patients with relapsed refractory B lymphoid malignancies in 2017 (1, 2). There are 6 FDA-approved CAR-T products (four CD19 CAR T-cell products and two B cell maturation [BCMA] CAR T-cells) for hematological malignancies; several are under investigation for other malignancies (1).

Clinical trials have demonstrated encouraging outcomes regarding hematological malignancy-related outcomes, albeit accompanied by infectious complications and toxicity-related adverse events (3). The burden of infection among CAR T-cell therapy recipients remains a critical consideration in managing these patients. Most patients are at high infection risk due to their underlying malignancies, prior lines of cancer-directed treatment, and pre-CAR T-cell lymphodepletion (2, 4, 5). Furthermore, the intensive lymphodepleting chemotherapy given before CAR-T infusion exacerbates immune system suppression, heightening susceptibility to opportunistic infections. Post infusion, immune dysregulation can lead to cytokine release syndrome (CRS), immune effector cell-associated neurotoxicity syndrome (ICANS), and hemophagocytic lymphohistiocytosis (HLH) -like syndrome; their treatments further predispose to infection (6–8). The post- CAR T-cell period is marked by varying degrees of lymphopenia, B-cell depletion, and hypogammaglobulinemia, which may influence long-term susceptibility to infection (5).

Prophylactic and monitoring measures based on an understanding of infection epidemiology are part of the efforts to lessen the burden of infections among CAR-T recipients. Due to the absence of comprehensive data, prophylactic and management strategies are based on consensus guidelines largely extrapolated from the post-hematopoietic stem cell transplant (HSCT) population (4, 9), and approaches vary widely by institution. This review aims to comprehensively analyze the epidemiology and risk factors associated with infections following CAR T-cell therapy in patients with hematological malignancy. It explores strategies for managing infectious risks associated with treatment-related toxicities and offers suggestions for screening, prophylaxis, immunoglobulin replacement therapy (IgRT), and vaccinations. Further research is needed in all these domains to expand our knowledge and optimize clinical practice.

## 2 Epidemiology of infections

The incidence of infection in patients receiving CAR T-cell therapy varies widely across prospective and retrospective trials, likely reflecting differences amongst patient populations, CAR T-cell related factors, definitions of infection, and follow-up duration. Epidemiology varies with CAR T-cell target and with time since cell infusion (day 0) due to the chronological evolution of infectious risk factors (4). For this review, epidemiology will be discussed in the context of two broad time points: those occurring in the early period, between days 0 and 30, and those occurring in the late period, beyond day 30. Overall, across the largest observational studies of infectious risk, infections were reported in 19–69% of patients after CD19-directed CAR T-cell therapy (2, 5), and in 42%-69% of patients after BCMA-directed CAR T-cell therapy; life-threatening infections are infrequently reported (2). A recent meta-analysis reported the pooled incidence of infection-related mortality as 1% (95% CI 0.01–0.02) and comparable amongst hematological malignancies (MM, ALL, NHL) (10).

### 2.1 Early infections: day 0 to day 30

Cohort studies evaluating infectious complications after CD19-directed CAR T-cell therapy report a higher incidence of infections within the first month and a subsequent decrease in the following months (11–20). The largest study of infectious complications after CD19-directed CAR T-cell therapy to date followed 133 patients with various malignancies (ALL, CLL, NHL) for up to 3 months after infusion; infection density was reported at 1.19 infections for every 100 days at risk within the first 28 days after cell infusion, and 0.67 between days 29 and 90 (21). The frequency of serious infection (grade 3 or higher) varies between 5–32% across the largest trials (2). Most documented early infections are bacterial, including both bacteremias and site infections; clostridium difficile colitis has specifically been identified in multiple trials (12, 13). The second most common infection is viral infections, of which respiratory viral infections account for the majority12-14. Hill et al. reported that infections, if present before lymphodepletion, can progress after CAR T-cell infusion (bacterial sinusitis, invasive fungal sinusitis, perirectal abscess) (11).

Patterns of early infection after BCMA-targeted CAR T-cell therapy differ somewhat from those encountered after CD19-directed therapy, likely reflecting differences in infectious risk attributable to patients’ baseline malignancies. Infectious risk is reported to be highest in the first 30–100 days and decreases thereafter (22, 23). Most infections are mild to moderate in severity and involve the respiratory tract. When a microbiologic diagnosis is made, bacterial and viral pathogens are identified, but viral etiologies are slightly more common (22, 23). Severe infections are infrequently reported; by one report, most serious infections and bacterial bloodstream infections occurred in the first 28 days after BCMA CAR-T infusion (23).

### 2.2 Late infections (beyond day 30)

CD19-directed CAR T-cell therapy results in the depletion of endogenous B-cells and hypogammaglobulinemia, which lasts for an unclear duration and may impact long-term risk for infectious complications (19, 24). Overall infection density decreased over time by one report; the incidence decreased from 11.7 infections per 1000 person-days in the first 30 days to 2.3 between days 31 and 90, and incidence continued to decrease over time (16). Cordeiro et al. reported infection density beyond day 90 was 0.55 infections/100 days at risk, or 2.08 per patient-year (25). The most common infections reported are of the respiratory tract; in some reports, viral etiologies predominate (13, 16, 21); in other reports, bacterial etiologies remain important causes of infection (12, 25). Bacteremias (21)and other bacterial site infections, particularly those of the urinary tract (12), also continue to be reported. In another cohort of 60 diffuse large b-cell lymphoma (DLBCL) patients, 37% of patients who developed bacterial infections after day 30 had documented bacterial infections between days 0 and 30 after CD19-directed therapy (12). Most infections are mild-moderate in severity and managed in the outpatient setting (12, 13, 16, 25).

The cumulative incidence of infection also declines over time among patients with multiple myeloma treated with BMCA-directed CAR-T therapy (23, 26). Bacterial infections continue to be reported between days 31 and 100, but viral infections predominate after that, mostly mild to moderate in severity (23, 26, 27). Amongst bacterial infections, site infections, specifically pneumonia and sinusitis, were most frequently reported in the late period by two reports (27, 28). The epidemiology of less common infections and risk factors are discussed later in the article.

Key points:

-The risk of infection is higher in the early risk period compared to the late risk period.
-Bacterial infections are relatively common during the neutropenic phase, then viral infections are more common. BCMA CAR-T recipients have a comparable incidence of viral infections to bacterial infections in the early risk period.
-Data on long-term infection incidence and risk is still evolving.

## 3 Factors associated with infectious risk

Factors influencing infection risk in CAR T-cell therapy recipients can relate to the host at baseline or the intervention; only a few known factors are modifiable. We will examine infectious risk in the context of two broad categories: pre-CAR T-cell infusion and CAR-T/post-CAR T-cell infusion. The post-CAR-T period can have early and late risk factors. Data comes from small, single-center experiences with heterogeneous cohorts and varying patterns of prophylaxis and management, which limits cross-trial comparisons. However, several distinct patterns do emerge.

### 3.1 Pre-CAR-T risk factors

CAR T-cell therapy recipients often are heavily treated and can have varying degrees of pre-existing cytopenia, decreased bone marrow reserve, and hypogammaglobulinemia. The burden of pretreatment (>3 prior lines) has been identified as a risk factor for infection regardless of baseline malignancy and CAR T-cell target (15, 17, 21, 26). History of allogeneic HCT was identified as a risk factor for early bacterial and viral infections in a cohort of 84 pediatric and young adult patients with R/R ALL (14). Impaired baseline performance status is associated with increased infectious risk after CAR T-cell therapy (12, 26). Finally, a history of infection 30–100 days before lymphodepletion has been associated with increased infectious risk post in the early period after cell therapy in multiple studies (12, 15, 26); Hill et al. reported that severe early infections present prior to lymphodepletion progressed after CAR T-cell infusion (11). Bridging chemotherapy, which is at times given for disease control during the CAR-T manufacturing period, was identified as a risk factor for severe infection before day 30 amongst 85 adults receiving CD19-targeted CAR T-cell therapy for DLBCL (16). This relationship was also noted by Kambhampati et al. in a cohort of 56 adults with MM undergoing BCMA-directed CAR T-cell therapy (26).

Underlying malignancy type has shown to play a role: a recent systematic review of 41 studies with 3199 patients receiving CAR-T therapy for hematological malignancy identified multiple myeloma patients as those at the highest risk for bacterial and viral infections as compared to those with ALL or NHL (29). Mikkilineni et al., in a retrospective analysis of 162 children and adults with a variety of malignancies treated with CAR T-cells directed against a variety of targets (CD19, CD22, GD2, BCMA), also identified those with multiple myeloma as the group with the highest risk for infection (15). Among CD19-directed CAR T-cell therapy, Hill et al. identified diagnosis of ALL as a risk factor for infectious complication within 90 days among 133 patients (ALL, NHL, CLL) (11).

Baseline hypogammaglobulinemia (IgG<400mg/dL) has been reported amongst CD19- and BCMA-directed CAR T-cell therapy recipients (11, 23–26). However, the impact of this characteristic on overall infectious risk is unclear. While some reports detect an association between baseline hypogammaglobulinemia and infectious risk after CD19-directed CAR T-cell therapy (12, 24), one large study did not (11). Moreover, baseline hypogammaglobulinemia has been reported in up to 88% of patients before receiving BCMA-directed therapy (23), and this has not been found to correlate with post-CAR-T infectious risk consistently (26).

Baseline neutropenia (absolute neutrophil count, ANC<500cell/mm^3^) has been shown to increase infectious risk in the early period after CAR T-cell therapy (11, 15, 18, 30). Recently, the CAR-HEMATOTOX (HT) score model evaluated baseline marrow reserve (platelets, hemoglobin, ANC) and baseline inflammatory markers (ferritin level and CRP) as predictors of post-CAR-T prolonged neutropenia and clinical outcomes, including infection, where high HT score was found to be associated with high risk of severe infection (30–32) **Table 1**. This tool might become helpful in identifying patients at high risk for post-CAR-T complications, including infections.

**Table 1:** CAR-HEMATOTOX (HT) Score Model for Pre CAR-T Risk Assessment (31).

Baseline Features	0 Points	1 Point	2 Points
Platelet Count	> 175,000/µl	75,000–175,000/µL	< 75,000/mL
Absolute Neutrophil Count (ANC)	> 1200/µl	< 1200/mL	–
Hemoglobin	> 9.0 g/dL	< 9.0 g/dL	–
C-reactive protein (CRP)	< 3.0 mg/dL	> 3.0 mg/dL	–
Ferritin	< 650 ng/mL	650 – 2000 ng/mL	> 2000 ng/mL

Low: 0–1 High ≥ 2.

Key points:

-Prior lines of therapy, history of allogeneic HCT, underlying malignancy, performance status, baseline hypogammaglobulinemia, pancytopenia, and inflammatory markers have been associated with increased baseline before CAR-T.
-Baseline risk factor assessment is important to identify high-risk patients before CAR-T infusion.

### 3.2 CAR-T/Post CAR-T related risk factors

Lymphodepletion (LD) before CAR T-cell infusion attenuates the immune response to CAR T-cells, allowing for robust engraftment and anti-tumor effect (33, 34). Cyclophosphamide with fludarabine has been associated with lower infectious risk than other lymphodepletion regimens (11, 24). Notably, early infections most often occur at times of neutropenia (13), which may be pre-existing or brought on by bridging chemotherapy and/or LD prior to CAR T-cell infusion.

A high CAR T-cell dose (2 x 10^7 cells/kg) is associated with an increased risk for infection (11). This may be due to the reported relationship between higher CAR T-cell dose and CRS development and severity. This study identified an increased hazard for infection with each increase in CRS severity category (11). Multiple other studies have demonstrated this relationship between CRS severity and infectious risk (13, 24, 35). Park et al. identified CRS of grade 3 or higher as an independent risk factor for infection (adjusted hazard ratio 2.67, p = .05), particularly with bloodstream infection (13). Still, this relationship has not been replicated in other studies (12).

Post CAR T-cell therapy, the use of steroids and tocilizumab in the management of CRS is not consistently shown to be associated with increased infection density. Hill et al. did not identify treatment with corticosteroids as a risk factor for infectious complications, and there was insufficient evidence to ascertain any correlation between tocilizumab duration or dose and risk for infectious complications (11). However, other studies have found that CRS, tocilizumab use, and corticosteroid use were associated with increased infectious risk after BCMA-directed CAR T-cell therapy (23) and in the first 30 days after CD19-directed CAR T-cell therapy (16). It is observed that most infections occur after the onset of CRS and do not appear to precipitate or exacerbate it (11). ICANS grade >2 has been identified as a risk factor for infection after CD19-directed therapy in multiple studies (11, 12, 16). The mechanisms by which CRS and ICANS may predispose to infection are unclear; pre-clinical studies have shown that chronic CAR signaling may induce early exhaustion of T cells, but there is currently no evidence to suggest that this results in clinically significant immune dysfunction (36). Also, severe CRS is associated with hematological toxicity, which can indirectly increase infection risk (37). While high-dose systemic corticosteroids are well known to predispose to infection (12, 15, 16, 19, 30), the effect of limited doses of tocilizumab and/or anakinra on overall infectious risk is less clear (38). Long-term treatment with tocilizumab has been shown to increase susceptibility to opportunistic infections in rheumatoid arthritis patients treated with these agents (39).

Hematological toxicity, also known as Immune Effector Cell-Associated Hematotoxicity (ICAHT), is recognized as an important toxicity attributable to CAR T-cell therapy regardless of target (40). Cytopenia persists beyond the immediate post-CAR-T phase, and count recovery often follows a nonlinear trajectory–intermittent recovery is often followed by subsequent dips (40). Patients can develop severe bone marrow aplasia, often refractory to growth factor support. ICAHT is divided into early (day 0–30) and late (day +30) based on the depth and duration of neutropenia (40). ICAHT has come to be recognized as a novel toxicity category of CAR T-cell therapy (40). A real-world experience applying the grading system to a cohort of 549 patients treated with BCMA- or CD19-directed CAR T-cells for refractory B-cell malignancies (MM, DLBCL, MCL) found that severe ICAHT was associated with a higher rate of severe infections and inferior survival outcomes (41). Among ICAHT, late neutropenia has been reported in multiple trials involving BCMA-directed and CD19-directed CAR T-cell therapy and can occur in a biphasic pattern with an intermediate recovery period (25, 26, 42, 43). In multiple studies, prolonged neutropenia due either to CAR-T-related factors or persistent disease has been shown to increase the risk for late infections (11, 26). CD4 lymphopenia (CD4 < 200 cell/mm^3^) is also frequently reported, lasting beyond day 30 (42) and up to 1 year (16) after CD19-directed CAR T-cell therapy and up to 9–12 months after BCMA-directed therapy (26). However, the impact of lymphopenia on overall infectious risk still needs further exploration. One study in a cohort that received CD19-directed therapy for DLBCL detected no relationship between CD4 and CD8 count at 30 days and infectious risk over 1 year (27). However, another study did detect a trend toward increased infectious risk with post-CAR-T lymphopenia after BCMA-directed therapy (22).

Hypogammaglobulinemia (IgG, <400mg/dL) affects 16–40% of patients before CAR T-cell therapy, and levels may decrease further after cell infusion and remain low for months or even years (11, 16, 20, 26). Amongst pediatric and young adult populations, CD19-directed therapy is associated with prolonged B-cell aplasia in up to two-thirds of patients and can persist for up to 5 years (44, 45). In a real-world experience, as many as half of patients received immunoglobulin replacement therapy (IGRT) for IgG<400 mg/dL in the post-CAR T-cell period (46). The reported severity and duration of hypogammaglobulinemia differs amongst adult populations receiving CD19-directed therapy; in one study, about half of patients had IgG >400mg/dL at the 1-year time point (26) after BCMA-directed CAR T-cell therapy, IgG<300 mg/dL in 70% of patients between 30–90 days and in 41% of patients after 1 year. Hill et al. reported that among 39 patients, 22 (56%) had a total IgG concentration <400 mg/dL at any time post CD19 CAR-T, and the cumulative incidence of an IgG concentration <400 mg/dL by 3 and 12 months post–CD19-CARTx was 36% and 60%, respectively (47). The significance of prolonged hypogammaglobulinemia on infectious risk remains unclear and may vary with CAR-T target. BCMA and CD19 are expressed on normal B cells at different stages of differentiation; CD19 is expressed on B cells at earlier stages and is lacking from long-lived plasma cells, which maintain stable concentrations of antigen-specific antibodies (48). Targeting CD19, therefore, leads to B-cell aplasia and hypogammaglobulinemia, but pathogen-specific IgG levels may be maintained. This was illustrated by the persistence of seroprotective levels of measles antibody independent of total immunoglobulin level after CD19-directed CAR T-cell therapy in one study (47). In agreement with this finding, evidence suggests that infectious risk may not correlate with lower IgG levels in this population (20, 47). In contrast, BCMA is expressed on plasma cells, so targeting this would be expected to lead to more severe hypogammaglobulinemia with a decline in pathogen-specific antibodies. Loss of immunoglobulin diversity and pathogen-specific immunity after BCMA-directed CAR T-cell therapy has been demonstrated in two cohort studies to date (22, 49). Kambhampati et al. also found a trend toward more infections during times of profound hypogammaglobulinemia after BCMA-directed therapy (22).

None of the risk variables listed above has an established attributable risk. The interaction and cumulative risk of the aforementioned variables may impact the patient’s overall infection risk. Thus, it is important to evaluate each situation carefully to optimize screening and preventive measures.

Key points:

- Risk factors associated with and after CAR-T infections include the type of lymphodepletion regimen, the dose of CAR T-cells, CRS post-infusion, use of systemic steroids, ICAHT, and hypogammaglobulinemia.
- Management of ICAHT and hypogammaglobulinemia management may modify infection risk after CAR-T infusion; prospective trials are needed to support this approach.

### 3.3 Multidrug-resistant organisms and antibiotic utilization

Antibiotic utilization post-CAR-T infection remains high (50), given that the most common infections in the early post-CAR-T period are bacterial, and most IEC-associated toxicities can present with fevers. The epidemiology of MDRO infections among CAR-T recipients is unknown; Yang J et al. showed poor 1-year clinical outcomes associated with Carbapenem-resistant organism infections among these patients (51). The utility of MDRO screening to assess MDRO colonization [as studied among HCT recipients (52)] and its impact on clinical outcomes still needs further investigation for CAR-T recipients. Similarly, antibiotic-associated microbiome dysbiosis has been associated with poor response to CAR-T therapy and increased toxicity (53, 54). These findings highlight the unmet need to study MDROs and antimicrobial stewardship in these complex settings.

### 3.4 Fungal infections and their risk factors

Despite multifactorial immune suppression, both mold and non-mold infections are infrequently reported as complications of CAR T-cell therapy (11, 55). Based upon cohort studies, epidemiology varies with CAR-T target, possibly due to unique pre- and post-CAR T-cell period features.

A review of published studies in 2021 by Garner et al. among CD-19-directed CAR T-cell recipients reported 1–10% incidence of yeast and 0–7% incidence of mold infections; most fungal occurred within the first 30 days and often represented breakthrough yeast infections in patients receiving fluconazole or echinocandin prophylaxis (56). Earlier, Hill et al. reported a 3% incidence of fungal infections between days 0 and 28 amongst 133 patients who all received fluconazole prophylaxis during the period of neutropenia after CD19-directed CAR T-cell therapy; all patients with fungal infections were reported to have been treated for CRS or ICANS with tocilizumab and/or corticosteroids (11). Incidence of fungal infections declined between days 28 and 90; late fungal infections were noted to have occurred in patients who had undergone prior allogeneic HSCT. Invasive mold infections were documented in both early and late periods but remain rare (11). This pattern of early-period fungemia while on echinocandin prophylaxis has been described by Park et al., and others have also found low-frequency invasive mold infections in both early and late periods (12, 13). More data is needed, but according to these results, it seems that a higher net burden of immunosuppression—development of CRS/ICANS (11, 16), HLH (35), treatment with tocilizumab or corticosteroids (11), other immunomodulating agents, and higher burden of prior treatment (>5 prior lines of therapy) (16), and history of HSCT (11)correlates with risk for fungal infection after CAR T-cell therapy.

While the low incidence of fungal infections reported in studies suggests the efficacy of antifungal prophylaxis (11–13), one large study reported a similarly low 2.9% incidence of invasive fungal infections at 1-year follow-up amongst 280 CD19 CAR T-cell patients with NHL who did not receive any antifungal prophylaxis (35). That cohort was also reported to have a high (41%) prevalence of severe delayed neutropenia, which may have been expected to increase susceptibility to fungal infection. Five of eight fungal infections reported occurred before day 100, including non-mold and mold infections. Of the three invasive mold infections reported, all were diagnosed by day 100 (35). Two of these infections occurred in patients with CLL and had received ibrutinib (35), which is an independent risk factor for mold infection (57, 58). Garner et al., in their review of invasive fungal disease, also noted that 73% of invasive mold infections occurred in patients with B-ALL or CLL (56). Studies to date have not been sufficiently powered to detect differences in risk for mold infection by different malignancy types. However, the underlying immune deficits associated with CLL and B-ALL and treatment with Bruton’s tyrosine kinase (BTK) inhibitors likely impact infectious risk in these patients (59, 60).

The epidemiology of fungal infections after BCMA-directed CAR T-cell therapy is less well-characterized. The overall incidence of fungal infection at 6 months of follow-up amongst patients who received some form of antifungal prophylaxis during their period of neutropenia has been reported at 4% (49) and 6% (22). Cumulative incidence declines with distance from the date of infusion (49), although one report recorded fungal infections, including invasive mold infections, occurred beyond 30 days after CAR T-cell infusion (11). Other reports also identified invasive mold infections in the early period (27, 49). High-grade CRS^2516^ and severe prolonged neutropenia (49) are possible risk factors for mold infections in the early and late periods, respectively.

### 3.5 Pneumocystis *jirovecii* pneumonia and its risk factors

PJP has been reported in patients beyond 3 months after CD19-directed CAR T-cell infusion and typically occurs after PJP prophylaxis has been discontinued (12, 35) or when prescribed prophylaxis has not been appropriately taken (35). According to one report, most cases of PJP occurred in patients with CD4 <200 cells/mm (35). A recent report of a real-world research network database showed that among 1107 Cd-19 CAR-T patients and 280 BCMA CAR-T patients, the incidence of PJP pneumonia was 1.7% and 1.4%, respectively. Patients who developed PJP had a higher likelihood of prior dexamethasone usage (65% versus 43%, p=0.02) and a reduced duration of trimethoprim/sulfamethoxazole (TMP/SMX) prophylaxis (median 9 weeks [range 1 to 45] versus 19 weeks [range 1 to 106], p=0.002. There was no difference in overall survival among patients with and without PJP (median 518 days vs not-reached, HR 1.61, 95%CI 0.91 to 2.88) (61). PJP incidence remains low likely related to widespread use of prophylaxis for a reported 3–6 months starting after neutrophil recovery and/or low rates of sustained CD4 lymphopenia among BCMA CAR-T, though further data is needed (22, 49).

### 3.6 Cytomegalovirus infection and its risk factors

The epidemiology of CMV infection after CAR T-cell therapy is poorly understood due to inconsistencies in routine surveillance practices. One prospective trial of 72 adult CMV seropositive patients receiving CD19-, CD20-, or BCMA-targeted CAR T-cell therapy identified a 27% (95% CI 16.8–38.2) cumulative incidence of CMV viremia (62). No end-organ disease was observed in that cohort, although 5 patients received preemptive therapy. BCMA-directed CAR T-cell therapy and corticosteroid use for >3 days were significantly associated with CMV reactivation (62). Another study reported a 10% incidence of CMV viremia among 61 BCMA CAR-T recipients in the first 6 months after cell infusion; end-organ involvement was uncommon but was reported in 3 cases, including gastrointestinal and possible lung involvement (63).

In one recent study, CMV accounted for 11% of all documented viral infections in the first year after CD19-directed therapy for DLBCL and was one of the most common viral infections in the study period (64). ICANS grade 3 or 4, CRS grade 3 or 4, anakinra use for treating CRS/ICANS, and higher cumulative doses of steroids within the first 30 days after cell infusion were all associated with CMV reactivation in this report.

In one large study by Marquez-Algaba et al. among 95 CMV-seropositive patients receiving CD19 CAR T-cell therapy for aggressive B cell lymphoma, 42(44%) patients had at least one positive serum CMV viral PCR; only 7 patients received preemptive antiviral treatment, and no CMV end-organ disease was reported (65). Dexamethasone treatment was the sole independent risk factor associated with CMV viremia > 1000 IU/mL in the study (65). In another cohort, amongst 133 patients with various B cell malignancies receiving CD19-directed therapy, there was one case of CMV pneumonia that occurred between days 29 and 90 in a patient with B-ALL (11). A recent meta-analysis identified an increased incidence of CMV reactivation in NHL patients; there were 39 cases in 949 patients, most of which occurred in the late period (10). Fareed et al. reported among 230 CD-19 CAR-T recipients, 10% developed clinically significant CMV infection. CMV infection was observed more among the female gender, with low ANC and monocyte count at day 30, grade 2 or higher CRS or ICANS requiring higher doses of steroids with higher mortality (66). These results demonstrate that CMV can cause disease in specific high-risk groups after CAR T-cell therapy. However, the significance of CMV viremia in the absence of end-organ disease still needs to be further explored.

### 3.7 Herpes simplex virus and herpes zoster virus infection and its risk factors

Reports of (HSV) and (VZV) reactivations after CAR T-cell therapy are infrequent, perhaps owing to the widespread use of antiviral prophylaxis. Reactivations have been reported after both CD19 and BCMA-directed therapies after discontinuing prophylaxis or in the setting of nonadherence with the prophylactic regimen (11, 13, 24, 28). However, delayed reactivations have also been reported despite the appropriate use of prophylaxis. There were two cases (3% incidence) of herpes zoster reactivation while on acyclovir prophylaxis in one study; both cases occurred after day 30 post-CAR T-cell infusion in patients who had received CD19 CAR T cells for DLBCL (11). A recent meta-analysis detected an increased signal for HSV/VZV reactivations in the late period in those with NHL (10). End-organ disease is not often reported to our knowledge; there was one reported case of fatal HSV pneumonia after BCMA-directed therapy in the setting of severe CRS and acyclovir resistance (67).

### 3.8 Human herpes virus 6 infection and its risk factors

Multiple cases of HHV-6 encephalitis in CAR-T recipients have been reported in the literature (19, 68–72). In one recent study, HHV-6 accounted for 8% of all documented viral infections in the first year after CD19-directed therapy for DLBCL (64). Several reported cases occurred in the context of mental status changes that initially responded to steroids but then relapsed and lasted beyond the typical time course of ICANS, prompting further infectious workup (68, 69). There has also been one case report of fatal HHV-6 myelitis following CD19-targeted CAR T-cell therapy in a patient who developed CRS and ICANS that initially responded to corticosteroids with subsequent development of ascending flaccid paralysis (73). Younger age, more prior lines of therapy, including allogeneic HSCT, and receipt of systemic corticosteroids may also increase susceptibility and lower the threshold for investigation (69, 71). Recently, Lareau et al. reported that HHV-6 can be reactivated among cultured T cells; implications and significance of this finding still need to be determined (74).

### 3.9 Adenovirus infections and its risk factors

There are no studies on the incidence of adenovirus infection in patients treated with CAR T-cells. Logue et al. reported one case of adenovirus viremia in a patient with DLBCL within 30 days of receiving CD19-directed CAR T-cell therapy. Still, the clinical significance of this finding was unclear (16). There are case reports of hemorrhagic cystitis cases associated with adenovirus infection (75). Further study is needed to elucidate the epidemiology and manifestations of adenovirus infection in the post-CAR T-cell period.

### 3.10 Polyomavirus (BKv) infection and its risk factors

There are anecdotal reports of BK virus infections and hemorrhagic cystitis after CD19-directed CAR T-cell therapy (11, 13). There are no reports of such infections associated with BCMA-directed CAR T-cell therapy. Case reports of late development of progressive multifocal leukoencephalopathy associated with JV virus have also been described (76, 77). Given the lack of conclusive data on the risk and impact of reactivation, the index of suspicion should be high in the context of hemorrhagic cystitis or atypical neurological symptoms with multifocal demyelination.

## 4 Prevention of infections

Preventive strategies can also be divided into two-time points, pre-CAR-T, and post-CAR-T, keeping the risk modifiable risk factors in mind. Screening and prophylaxis strategies are determined before CAR T-cell therapy. In contrast, post- CAR-T strategies are focused on reducing infection risk by infection surveillance when needed, ongoing chemoprophylaxis, immunoglobulin replacement therapy (IGRT), and vaccination administration.

### 4.1 Baseline screening pre CAR T-cell therapy

Infection screening before CAR T-cell therapy is an important risk assessment component. HIV, HBV, and HCV serologic testing with reflex nucleic acid testing is recommended for all patients (4). False positive HIV nucleic acid amplification tests (NAAT) may be seen in CAR-T recipients when the CAR-T product was generated using lentiviral vectors, owing to the use of conserved regions of HIV-1 (78). Products prepared with murine gamma-retroviral vectors are not thought to carry the same risk. In the case of a true false positive test, confirmatory fourth-generation HIV-1/2 antibody and p24 antigen testing returns negative, and HIV-1 RNA may be detectable at a low level, possibly due to circulating cell-free DNA (78). No further intervention or monitoring is required in these cases, and CAR T-cell therapy may proceed as planned (78). Patients with HIV infection were excluded from clinical trials, so the safety of CAR T-cell therapy in the HIV-positive population is poorly understood (79). There are, however, published reports of successful treatment of HIV-infected patients with DLBCL with CD19-targeted CAR T-cell therapy (80, 81).

Patients with HCV viremia (chronic HCV infection) should be considered for antiviral treatment if compatible with liver function and overall clinical situation (82). If liver test abnormalities develop during treatment, then the potential role of HCV and other hepatotropic viruses should be assessed. Notably, hepatitis E has been reported to cause chronic disease in immunocompromised patients (83). Prophylaxis and surveillance strategies in the case of positive hepatitis B serologies are discussed in detail in the next section. Serologic screening for HSV1/2 and VZV is recommended in those not already receiving antiviral prophylaxis; this is also important for future consideration of VZV vaccination (4). Screening for Mycobacterium tuberculosis (MTB) should be considered in patients with risk factors for exposure (84). Tocilizumab use is independently associated with an increased risk for MTB infections in rheumatoid arthritis patients [91]; however, the impact of the short-term dosing utilized in CRS management has not yet been reported. Baseline toxoplasma serologies may be considered on a case-by-case basis (4, 9). Finally, screening for antibodies to Strongyloides stercoralis or empiric treatment with ivermectin should be considered in patients with a history of time spent in tropical or subtropical regions, given the risk for reactivation due to high-dose corticosteroids and/or tocilizumab (4, 9).

A complete history and physical exam should be performed to evaluate for active infections before lymphodepletion. If the evaluation concerns an infection, a more directed workup should be performed as indicated by the clinical situation.

### 4.2 Prophylaxis and monitoring post CAR T-Cell infusion:

Based on the current literature, the recommendations below represent our opinion; further prospective studies are needed to optimize these practices.

The role of antibacterial prophylaxis in this population is unclear; fluoroquinolones are often employed, but practice patterns vary widely across institutions. A large retrospective analysis of patients with DLBCL receiving CD19 CAR T-cells identified a significant reduction of severe bacterial infections with fluoroquinolone prophylaxis in patients who were categorized as CAR-HEMATOTOX^high^ but not in those who were CAR-HEMATOTOX^low^ at baseline (41). A risk-adapted approach may be the optimal strategy, but more studies are needed, and institutional guidelines vary. Multiple studies have reported early bacterial infections with gram-negative organisms with acquired or intrinsic fluoroquinolone resistance, regardless of fluoroquinolone prophylaxis patterns (11, 13). It is unclear how much antibacterial prophylaxis strategies should be adjusted to account for this. We suggest using fluoroquinolone prophylaxis when ANC<500 cells/mm^3^. Optimal infection prophylaxis strategies for those with severe ICAHT require further study. There may be a role in closely monitoring patients with high CAR-HEMATOTOX scores before lymphodepletion or among those who develop severe ICAHT post-CAR T-cell therapy (41).

The role of routine antifungal prophylaxis is also debated, given the low reported incidences of non-mold infections with or without prophylaxis (5, 11, 13, 16, 35, 49, 85). Until further data are available, we recommend fluconazole prophylaxis during periods of neutropenia in all CAR T-cell recipients. Anti-mold prophylaxis should be considered in select high-risk patients for invasive mold infection. We would recommend the use of a mold-active azole in those with a history of invasive fungal infection (IFI), ANC < 500 cell/mm3 for >21 days present prior to CAR T-cell therapy or developing after infusion, and treatment with high-dose steroids and for longer duration. Duration of anti-mold prophylaxis should be determined on a case-by-case basis; in the presence of multiple risk factors, extending prophylaxis beyond ANC recovery (ANC > 500 cell/mm^3^ for 3 consecutive days) may be appropriate.

PJP prophylaxis has also been widely adopted, though opinions on the optimal duration of prophylaxis remain mixed. Given reports of PJP infections diagnosed beyond 3–6 months after cell infusion in the context of prolonged CD4 lymphopenia and prophylaxis discontinuation or incomplete adherence (5, 12, 35), we would suggest starting prophylaxis upon initiation of lymphodepleting chemotherapy and continuing for at least 6 months or until CD4 count >200 cell/mm^3^. HSV/VZV prophylaxis with acyclovir or valacyclovir has been widely adopted and recommended.

Entecavir is recommended when HBsAg is positive and/or when HBV DNA is detectable before receiving CAR T therapy and should be continued for at least 6–12 months from infusion due to the risk of reactivation following B cell depletion (4, 9). If anti-HBc is positive but surface antigen and DNA are negative, antiviral prophylaxis OR lab monitoring with liver function tests and HBV DNA every 1–3 months can be performed. Those with anti-HBc+ and anti-HBs+ likely have a lower risk of reactivation. A more conservative approach may be appropriate in multiple myeloma patients given their more severe humoral immunodeficiency, including pathogen-specific immunoglobulin deficiency, which is depleted after BCMA CAR T-cell therapy (49). **Table 2** summarizes recommendations for antimicrobial prophylaxis.

**Table 2:** Proposed Antimicrobial Prophylaxis for CAR-T Patients (4, 86–90).

	Agent	Alternative agent (s)	Comment
Antibacterial	Levofloxacin		Start when ANC < 500 and continue until neutrophil recovery (ANC >500 for at least 3 days)
Antifungal	Fluconazole	Micafungin	Start when ANC<500 and continue until neutrophil recovery (ANC >500 for at least 3 days)
Anti-mold	Voriconazole	Posaconazole	Consider in those at high risk for mold infection: ANC<500 for >21 days, treatment with prednisone >20mg for >2 weeks or equivalent, history of IFI, history of allogeneic HSCT; duration determined case by case
Anti-PJP	Trimethoprilm/ Sulfamethoxazole (TMP/SMX)	Inhaled pentamidine OR dapsone OR atovaquone	Start with lymphodepleting chemotherapy and continue for at least 6 months post- CAR T infusion or until CD4 count >200 cell/mm^3^
Antiviral	Acyclovir	Valacyclovir	Start with lymphodepleting chemotherapy and continue for 6–12 months or until CD4 >200 cell/mm^3^

### 4.3 CMV monitoring, prophylaxis, and pre-emptive therapy post CAR-T infusion

The role of CMV monitoring remains a topic of debate. Still, there are reports of both CMV viremia and end-organ disease occurring after CD19- and BCMA-directed CAR T-cell therapy (11, 28, 65, 91). We suggest checking baseline CMV serostatus and serum PCR in all patients proceeding with CAR T-cell therapy. Those with a negative CMV assessment before CAR T-cell infusion and who do not require systemic steroids for related complications would likely not benefit from regular monitoring.

Those with a positive CMV assessment and/or those who require >3 days of high-dose systemic steroids and have received BCMA-directed therapy may benefit from weekly CMV monitoring during the first 6 weeks after CAR T-cell therapy. This recommendation is based upon limited data on the kinetics of CMV reactivation after CAR T-cell therapy (62), the duration of monitoring should be adjusted on a case-by-case basis. There is insufficient data to suggest prophylactic strategies in this setting. The optimal role and threshold for pre-emptive therapy also remain unclear and must be balanced against the risks of therapy-related toxicity, including further bone marrow suppression.

Allogeneic CAR T-cell therapy (allo CAR-T) is an active research area and may carry unique risk factors for CMV and other viral reactivations. Chemotherapy ahead of allo CAR-T, or lymphodepletion in preparation, may require novel strategies such as using alemtuzumab to mitigate the risk of GVHD and promote engraftment and expansion of the infused cells (92, 93). The resultant deeper and more prolonged lymphodepletion would pose an important risk factor for viral reactivation (93).

## 5 CRS, ICANS, and fever after CAR-T infusion

CAR T-cell infusion can result in CRS, manifesting as fever, capillary leak, and end-organ dysfunction (94–96). The typical time frame for CRS presentation is 2–7 days after CAR T-cell infusion, but it may occur within hours or up to 10–15 days after infusion (97). ASTCT consensus grading ranges from grade 1 to 5, reflecting a spectrum of presentations from fever and constitutional symptoms to critical illness requiring invasive monitoring, pressors, and ventilatory support (97). ICANS is regarded as a separate clinical entity characterized by encephalopathy that can be progressive, language disturbances, motor weakness, seizures, and cerebral edema (3, 95, 96). ICANS typically present later than CRS, with a typical time to onset of 4 to 19 days after receiving CAR T-cell infusion (98). While the two entities do not always occur in the same host, severe ICANS is unlikely to be seen without severe CRS (95, 96).

Both CRS and ICANS can mimic infections. Severe CRS may present as septic shock requiring invasive monitoring and is managed with high doses of steroids; critical illness and steroids can cause neurologic symptoms (3, 95–97, 99), which may be difficult to distinguish from ICANS. Moreover, managing CRS and ICANS involves multimodal systemic immunosuppression, which can mask the clinical presentation of infection. Corticosteroids and anti-IL-6 therapy with tocilizumab remain cornerstones of CRS management, while corticosteroids are the treatment of choice for ICANS with the addition of anti-IL-1 therapy with anakinra in refractory cases (3, 94–96). More recently, there has been a paradigm shift towards pre-emptive use of these modalities to prevent progression to higher-grade manifestations (98, 100).

Given the ambiguity of the clinical presentation, it is crucial to initiate broad-spectrum antibiotics promptly and to investigate reversible infectious causes as part of the initial assessment. In practice, broad-spectrum antibiotics are generally administered in the setting of CRS after CAR-T infusion in the setting of febrile neutropenia, hemodynamic instability, and hypoxia (4, 8). Empiric coverage should consider the patient’s history of prophylaxis, and local antibiogram and infection work-up should be initiated. Infectious disease consultation should be considered early, especially when the clinical picture is complex. There is increasing awareness of the adverse effects of broad-spectrum antimicrobials, including microbiome dysbiosis, the emergence of multidrug resistance organisms, and Clostridium difficile infection (101, 102). To mitigate these risks, we advocate for diligent de-escalation strategies. Consideration should be given to stopping broad-spectrum antibiotics in patients with neutropenia who have been afebrile for 72 hours and remain without clinical or microbiologic source of infection (103–105).

## 6 Emergent hemophagocytic lymphohistiocytosis-like toxicities and infections

Immune effector cell-associated HLH-like syndrome (IEC-HS) has been described post-CD-19 directed CAR-T infusion, with severe and fulminant cases occurring in <1% of patients (6). Earlier reports characterized IEC-HS progressing from cases of severe CRS, but recently, there has been increasing recognition of delayed HLH-like toxicities (7). IEC-HS is also becoming more apparent after BCMA CAR T-cell therapy (105). The definition of IEC-HS is outside the scope of this paper; it is important to recognize that the clinical presentation can mimic infection and that infection can co-occur with this entity. We recommend initiating appropriate antimicrobials while infectious work-up is underway. Treatment of IEC-HS involves extensive immunosuppression, thus increasing overall infection risk. **Table 3** highlights the immunosuppressive agents and the infections associated with them. As treatment strategies for CRS, ICANS, and IEC-HS evolve, it is imperative to keep the infectious risks attributed to these agents in mind and consider adjusting the prophylaxis strategy to account for their use.

**Table 3:** Immunosuppressive Agents Used for Treatment Of CRS/ICANS/HLH and their Associated Infection.

Immunosuppressive Therapy	Associated Infection Risk	Prophylaxis Strategy Considerations
Steroids (dexamethasone, methylprednisolone)	Well-known association with fungal infections, viral reactivations, and PJP, which is also noted in some cohorts after CAR T-cell therapy (11, 16, 22, 35, 49, 65)	Mold active prophylaxis HSV/VZV prophylaxis if seropositive Weekly monitoring for CMV reactivation and strong consideration of pre-emptive therapy
IL-1 Receptor antagonist (anakinra)	Well tolerated with extended treatment in the rheumatoid arthritis population (106); no specific association with infectious risk in limited experience after CAR-T (107) In combination with steroids, the risk of infection may be higher (106)	If being administered with steroids, above considerations apply
IL-6 receptor antagonist (tocilizumab, siltuximab)	Safety profile post CAR-T infusion is unclear; in one report, use was associated with infections and death (108). In other populations, it has been associated with tuberculosis (TB), other mycobacterial infections, and fungal infections (39)	Mold active prophylaxis HSV prophylaxis if seropositive CMV preemptive therapy Weekly monitoring for viral reactivation Bacterial prophylaxis when ANC<500
JAK1/2 inhibitor (ruxolitinib)	Safety in CAR T-cell population unclear; associated with higher rates of VZV infection and hepatitis B reactivation (109, 110) in hematological malignancy population; also reports of disseminated TB, cryptococcal infection, toxoplasmosis, CMV disease, mold infections (109, 111).	Mold-active prophylaxis (with attention to drug-drug interactions between ruxolitiib and azoles) PJP prophylaxis VZV prophylaxis if seropositive Weekly monitoring for CMV reactivation among those on multiple and strong consideration of pre-emptive therapy HBV prophylaxis if HBSAg+ and/or HBV DNA PCR is detectable Bacterial prophylaxis when ANC<500
Chemotherapy (etoposide)	Bacterial infections with neutropenia	Attention to bacterial prophylaxis when ANC<500
Anti-IFN-gamma monoclonal antibody (emapalumab)	Viral reactivations, fungal infections, TB reactivation and other mycobacterial infections have been reported in other populations (112, 113)	Fungal prophylaxis on case by case basis PJP prophylaxis VZV prophylaxis if seropositive Weekly monitoring for CMV reactivation and consideration of pre-emptive therapy

## 7 Post-infusion hypogammaglobulinemia and IgRT

Although prophylactic IgG has received regulatory approval in specific immunocompromised groups, less extensive data indicates similar effectiveness in CAR T-cell recipients^5510^. IgRT has primarily been established as beneficial in preventing serious bacterial infections; there is less evidence to support its use in the prevention of viral infections, which are more frequently reported as late complications of CAR T-cell therapy (48). In fact, studies to date have failed to reliably show an association between hypogammaglobulinemia and infectious risk after CAR T-cell therapy regardless of targe (47, 48), and there is similarly mixed data on the efficacy of IgRT in these populations (22).

However, based on the pathophysiology of humoral immunodeficiency after CAR T-cell infusion, there is consensus that IgG levels should be monitored both pre-CAR T-cell infusion and monthly for at least 3 months after infusion (4, 5). A threshold IgG level of 400 mg/dL is frequently used to initiate IGRT in adults (4, 5). Adverse events are infrequently reported after IVIG infusion but do include mild and occasionally severe infusion reactions, and delayed toxicities including thrombosis that may manifest as ischemic stroke or myocardial infarction, renal failure, and transient hemolytic anemia (48, 114). IVIG is also associated with significant costs and accessibility issues, so use should be judicious and guided by a multidisciplinary team effort (48).

Given mixed data on the impact of hypogammaglobulinemia on infectious risk after CD19-directed CAR T-cell therapy, as well as the preservation of pathogen-specific antibodies detected after CD19-directed therapy, we would suggest a tailored approach to IgRT in this subset of CAR-T recipients. It may be appropriate to reserve IgRT for patients with recurrent and/or severe infections and IgG < 400 mg/dL before cell infusion and in the first 3–6 months after CD19-targeted therapy. In those with IgG levels between 400 mg/dL and 600 mg/dL, IGRT may also be considered for severe or recurrent infections. If there are recurrent infections in normal serum immunoglobulin levels, testing for functional humoral immune dysfunction, e.g., vaccine response, may be helpful (4, 114). A more conservative approach may be appropriate in BCMA CAR T-cell recipients given their more profound depletion of pathogen-specific immunity (22, 49); IgRT may be considered in those with IgG< 400 mg/dL, even without evidence of recurrent infection with caution (48). In these patients, distinguishing between normal IgG and paraprotein with serum protein electrophoresis is important. Prospective trials are needed to optimize these strategies.

## 8 Vaccinations for CAR T-cell recipients

B-cell aplasia and hypogammaglobulinemia can increase susceptibility to infection by vaccine-preventable encapsulated bacteria such as *Haemophilus influenzae* type B, *Neisseria meningitidis*, and *Streptococcus pneumoniae*. Appropriate vaccination has the potential to prevent infections, decrease their severity, mitigate the need for IgRT, and improve survival and quality of life (4).

Walti et al. identified decreased seroprotection levels against *S. pneumoniae and H. influenzae* type B after CD19-directed therapy (115). In this same study, levels of IgG against measles, tetanus toxin, Epstein-Barr virus, varicella-zoster virus, and herpes simplex virus remained detectable despite decreased total serum IgG and B-cell aplasia (115). Among those who attained CR, the proportion of participants with seroprotective IgG titers to vaccine-preventable infections was comparable to population-based seroprevalence data without re-vaccination (115). In contrast, a small cross-sectional study suggested that BCMA CAR T-cell recipients are less likely to have seroprotective IgG titers to vaccine-preventable infections (49). It remains unclear whether this reflects CAR T-cell therapy’s effects or indicates baseline humoral immunodeficiency; in that same study, measles-specific IgG was present in only 16% of patients before cell infusion (49).

The optimal timing of vaccination after CAR T-cell therapy is unclear, as the timing of cellular and humoral immunity reconstitution after CAR T-cell therapy can vary widely (2, 9). However, a recent study reported that neither B-cell aplasia nor hypogammaglobulinemia reduced influenza vaccine immunogenicity after CAR T-cell therapy (116), suggesting that strictly following these markers to assess for immune reconstitution may create unnecessary delays in vaccination. Another study noted recovery of seroprotective measles IgG level by 114 days after CD19 CAR T-cell therapy without vaccination and coinciding with CD19+ B-cell recovery (47), suggesting that immune recovery may obviate the need for aggressive re-vaccination strategies in select groups. Optimal strategies may need to be considered based on the CAR-T target.

Vaccination against influenza during flu season should be considered at least two weeks before lymphodepletion; additional vaccination before cell infusion is likely of low utility given the impending severe immunosuppression. Clinical practice for revaccination currently follows protocols used in HCT recipients, though the need for revaccination for all previously completed vaccine series remains unclear (4, 48, 117, 118). In general, for patients who are in remission and not planned to receive further T-cell and/or B-cell depleting therapies, killed/inactivated vaccinations should be considered starting at least 6 months after CAR T-cell infusion, and live and adjuvant vaccines should be considered at least 1 year after cell infusion. IgRT may interfere with the efficacy of live vaccines, so these should generally be delayed for at least 9 months after the most recent IgRT (4, 48). This recommendation is based on the guidelines for vaccination of immunocompromised hosts (118, 119) and the kinetics of immune reconstitution after CAR T-cell therapy (11, 120, 121). SARs-CoV-2 vaccination can be started after 90 days given (122).

Based on the epidemiology of infections after CAR T-cell therapy and known effects of B-cell aplasia and hypogammaglobulinemia, key vaccines to consider include annual influenza, *Streptococcus pneumoniae*, *Haemophilus influenzae type b*, *Corynebacterium diphtheriae* and *Clostridium tetani* toxins, *Bordetella pertussis*, and hepatitis A and B viruses. For patients 50 years old who are seropositive for VZV or have a history of shingles, recombinant zoster vaccine (Shingrix) should also be considered. Conjugated vaccines should be used, when possible, given higher response rates in immunocompromised patients (123). Measuring vaccine responses may be helpful to assess the utility of additional vaccination on a case-by-case basis (4).

For respiratory viruses, COVID-19 and RSV vaccines (age-dependent indication) should also be considered in addition to the influenza vaccine (124, 125). Data regarding the durability of seroprotection provided by pre-infusion COVID vaccination and the immunogenicity of mRNA-based COVID vaccines after CAR T-cell therapy are mixed (126). We suggest following the CDC’s guidance on COVID-19 vaccines for moderately and severely immunocompromised people (127). The efficacy of RSV vaccines in CAR-T recipients is unknown. **Table 4** highlights the vaccine recommendations for CAR-T recipients.

**Table 4:** Vaccines For CAR T-cell Therapy Recipients.

	Killed/inactivated vaccines	Live and non-live adjuvant vaccines
Eligibility	6 months post-CAR-T 2 months since last IGRT	1-year post-CAR-T
Contraindications	• IGRT within the past 2 months • Receiving T-cell or B-cell directed immunosuppressive therapy. • Receipt of anti-CD20 or anti-CD19 in the prior 6 months • Actively receiving chemotherapy	• Received anti-CD19 or anti-CD20 therapy within the past 6 months. • 1 year post CAR T-cell therapy • 2 years post autologous or allogeneic HCT • <1 y off of all systemic immunosuppressive therapy • < 8 months after the last dose of IGRT • Absolute CD4 count < 200 cells/mm^3^ • Absolute CD19+ or CD20+ B cell count < 20 cells/mm^3^ • Actively receiving chemotherapy
Vaccinations to consider	Influenza Covid-19 Pneumococcal conjugate Pneumococcal polysaccharide Diphtheria, tetanus, and acellular pertussis (DTaP) Hepatitis A virus Hepatitis B virus	Varicella Zoster Virus

*Those who have undergone prior HCT without completing all post-transplant re-vaccinations should restart the whole vaccination series once they meet the eligibility criteria described above and all HCT-related criteria. Antibody responses can be checked if possible before and after starting the vaccine series to guide clinical decision-making; if there is no response to vaccination despite meeting eligibility criteria, then further vaccinations can be attempted once there is immune reconstitution (IgA > 6mgdL + CD19 or CD20 B cell count > 20cells/mm^3^ + CD4 count > 200 cells/mm^3^).

*Those post-HCT who have completed their post-HCT vaccination series OR those who have never undergone HCT should also be fully vaccinated once they meet the eligibility criteria described above. Antibody titers can be monitored before and after vaccination to guide subsequent steps in the vaccine series. This allows for preserved immunity and the ability to generate a boosted response with a single dose of a given vaccine.

*Antibody response can be determined by checking serum IgG titers to S pneumoniae (23 serotypes), tetanus toxoid, hepatitis A virus, and Hepatitis B virus surface antigen.

*For non–S pneumoniae vaccines, a response is defined as at least a twofold increase in IgG from prevaccination to 1 to 2 months postvaccination or achieving a seroprotective IgG level at 1 to 2 months postvaccination. For the S pneumoniae vaccine (Prevnar 15 or 20) response is defined as at least a twofold increase in IgG from prevaccination to 1-month postvaccination, achieving an IgG ≥ 1.3 ug/mL for ≥50% of the Prevnar serotypes, or as defined by the testing laboratory.

## 9 Future directions and knowledge gaps

The field of CAR T-cell therapy is continuously evolving, with the development of newer targets, combination therapies, newer CAR-T designs, and off-the-shelf products [121], and important gaps remain in our understanding of short and long-term infectious complications. As new CAR T-cell targets are introduced to the market for hematological malignancies as well as for solid tumors, and potentially for use in autoimmune diseases and suppression of rejection in organ transplant (82), transparency and completeness of reporting about infectious complications of these therapies will remain critical as we seek to devise strategies to mitigate risks (128). An example is the development of allogeneic (off-the-shelf) CAR T-cell therapy; these products come with the risk of rejection and development of graft versus host disease (GVHD) and may require unique pre-infusion LD and post-infusion immunosuppression strategies (129). Allogeneic T cell-based products have entered Phase I and II clinical trials, and different effector cell types, such as NK cells and macrophages, are also under investigation (129). Dual-targeted CAR T-cells, for example, targeting CD19 and CD22 for treating R/R aggressive B-cell lymphomas, are also in development (130). CAR-T therapy for Acute Myeloid Leukemia can lead to myeloablation (131). Understanding infection incidence and risk associated with these novel treatments is vital to developing mitigating strategies.

The infection burden of DNA viruses post CAR T-cell therapy is still evolving (63, 74, 132, 133), and might vary among CAR T-cell targets. Prospective studies are important to understand CAR T-cell recipients’ specific pathogen and infection burden. Similarly, long-term infection data are lacking. Extended research is necessary to evaluate the frequency of late-onset infections and the influence of persistent immune dysfunction on the risk of infections among individuals treated with CAR T-cell therapy.

Microbiome dysbiosis among CAR T-cell recipients is associated with poor clinical outcomes, including CAR-T toxicities and response to therapy (53). Similarly, a non-antibiotic-disrupted gut microbiome is associated with improved clinical response to CD-19 CAR T-cell therapy (134). This highlights the importance of developing strategies for early identification of infection mimickers post CAR T-cell and prompt de-escalation of broad-spectrum antimicrobials when appropriate.

In conclusion, infections remain an important concern among CAR T-cell recipients, and our understanding of infection dynamics among different settings is still evolving. Addressing these knowledge gaps is imperative to improving patient outcomes. Additionally, ongoing research efforts should prioritize the establishment of standardized guidelines for monitoring infections, implementing prophylactic measures, and administering treatments that cater to the unique requirements of CAR T-cell recipients. This approach will ultimately enhance our patients’ overall quality of care and prognosis.

## Author contributions

ZS: Conceptualization, Methodology, Supervision, Writing – original draft, Writing – review & editing. SA: Data curation, Writing – original draft, Writing – review & editing.
